# Effects of low-intensity home-based exercise on cognition in older persons with mild cognitive impairment: a direct comparison of aerobic versus resistance exercises using a randomized controlled trial design

**DOI:** 10.3389/fmed.2024.1392429

**Published:** 2024-06-21

**Authors:** Kitsana Krootnark, Nithinun Chaikeeree, Vitoon Saengsirisuwan, Rumpa Boonsinsukh

**Affiliations:** ^1^Department of Physical Therapy, Faculty of Physical Therapy, Srinakharinwirot University, Nakhon Nayok, Thailand; ^2^Department of Physiology, Faculty of Science, Mahidol University, Bangkok, Thailand

**Keywords:** light exercise, executive function, attention, memory, aging

## Abstract

**Background:**

It has been reported that both aerobic exercise and resistance exercise can improve cognitive function in older people with mild cognitive impairment (MCI), but it is unclear which type of exercise has a higher impact on cognitive function. Additionally, low-intensity exercise is considered safe for the elderly and can be done at home. This study aimed to compare the effects of 3-month low-intensity home-based exercises, aerobic versus resistance exercises, on cognitive function in people with MCI.

**Methods:**

This study was a single-blind randomized controlled trial conducted in a suburban community. Ninety eligible participants aged 60–80 years were randomly assigned into aerobic exercise, resistance exercise or control group (30 in each group). The aerobic and resistance exercise groups underwent 3 months of low-intensity exercise at home (35 min/day, 5 days/week). The control group performed their usual daily activities. The Montreal Cognitive Assessment Thai version (MoCA), Trail Making Test Part A and B (TMT-A, TMT-B), Stroop Color and Word Test (SCWT), forward and backward Digit Span Test (DST-F, DST-B) and Stick Design Test (SDT) were administered before training, 3-month after training and 3-month follow-up.

**Results:**

All participants completed a 3-month exercise program, but during the follow-up, data were gathered from 28, 27, and 26 participants in the aerobic, resistance, and control groups, respectively. Both aerobic and resistance groups showed significant improvements in all outcome measures during posttraining and follow-up, except SDT, while there was no cognitive improvement in control group at posttraining and follow-up. Compared to those in the control group, the aerobic group had significant improvements in MoCA, TMT-A, TMT-B, and SCWT, while resistance group had significant improvements in MoCA and TMT-B at posttraining and follow-up. There were no differences in any outcome measures between aerobic and resistance groups, except SCWT, which was significantly greater in the aerobic group than in the resistance group posttraining.

**Conclusion:**

Low-intensity exercise, whether aerobic or resistance training, was effective at improving cognitive function in older people with MCI, and the effects were sustained at the 3-month follow-up.

**Clinical trial registration:**thaiclinicaltrials.org, TCTR20231110003.

## 1 Introduction

Mild cognitive impairment (MCI) refers to a state in which cognitive function declines more than normal relative to chronological age. MCI is characterized by impaired brain function in one or more of five cognitive domains: executive function, complex attention, learning and memory, perceptual-motor function, and language. The most common early symptom of older adults with MCI is decreased memory performance. Another impairment that can be found in this population is in executive function, specifically working memory, inhibitory control and mental flexibility, which are related to the ability to organize, manage, and perform daily activities (1). Older persons with MCI experience a decline in cognition or performance on cognitive tasks, but these symptoms have no impact on their ability to perform basic daily activities (2). The worldwide prevalence of MCI in people aged 50 years and older is more than 15% (3), and the incidence of MCI per 1,000 person-years is more than 22.5 for people aged 75 years and older (4). Research related to preventing dementia or treating cognitive impairment is therefore becoming increasingly necessary.

Physical activity is known to be positively associated to cognition, especially in executive function domain (5, 6). It is also a modifiable risk factor to reduce or prevent cognitive decline that comes with aging (7). Physical exercise is a subset of physical activity that has been used to prevent cognitive impairment. Evidence from systematic reviews of physical exercise in older people with MCI demonstrated that physical exercise was effective at improving cognitive function, namely, working memory, in the domains of global cognition and executive function (8–10). One of the most popular forms of exercise for enhancing cognitive function is aerobic exercise, especially moderate-intensity exercise (10–12). Aerobic exercise programs targeted for this purpose usually include cycling and treadmill walking (13–15). The advantage of both types of exercise is that the intensity can be easily controlled and changed as needed, but treadmill walking and cycling require specific equipment, which may limit accessibility for seniors. Resistance training has also been demonstrated to enhance cognitive performance. Systematic reviews have indicated that resistance training improved global cognitive function and executive function domains, in terms of inhibitory control, in older people (16, 17).

Despite a systematic review on the benefits of resistance and aerobic exercise for older people with MCI, it is unclear which type of exercise, between aerobic and resistance exercise, has a greater impact on cognitive performance in older persons with MCI. Most studies have focused on the benefits of each type of exercise on cognitive function separately, without side-by-side comparisons (18–20). This information will be crucial for designing exercise programs to enhance cognitive function in older persons with MCI who may have restrictions or limitations on either aerobic or resistance exercise.

Low-intensity exercise, such as walking, lifting weights, and wall push-ups, is another type of exercise that can be easily performed by older people to reduce the risk of injury and can be performed at home. Home-based exercise is regarded as safe to perform alone and does not require any specific equipment or the use of household items. Studies on home-based exercise with multimodal exercise have been conducted for individuals with MCI, and the outcomes have focused only ever on functional performance outcomes (21, 22). There is no evidence that any type of home-based exercise can help older people with MCI enhance cognitive function.

Therefore, the aim of this study was to investigate the effect of low-intensity home-based exercise on cognitive function, specifically executive function, attention, memory, and perceptual-motor function domains, in people with MCI by directly comparing three groups, the aerobic exercise, resistance exercise and control groups. Cognitive function was assessed before training, at 3 months after training and at 3 months of follow-up. We hypothesized that cognitive function would be significantly improved in people with MCI who perform low-intensity home-based exercise compared to a control group. We also expected that the aerobic exercise group would experience greater improvement in cognitive function than the resistance exercise group.

## 2 Materials and methods

### 2.1 Participants

Ninety older people with probable MCI aged more than 60 years, both male and female, living in the suburban community participated. Sample size calculation determined 80% power, alpha of 0.05 and effect size of 0.3 (23), which assumed a total of 90 participants (30 participants per group) including 20% dropout. All participants had scores on the Montreal Cognitive Assessment (MoCA) of 17–24 points (24); able to stand and walk independently (with or without an assistive device); and able to follow verbal instructions. Participants were excluded if they met the following exclusion criteria: (1) diagnosed with dementia; (2) had a history or diagnosis of neurological diseases such as stroke or traumatic brain injury; (3) had a history or diagnosis of cardiovascular diseases such as myocardial infarction; (4) had a resting blood pressure > 160/100 mmHg; (5) had severe musculoskeletal conditions that interfered or limited compliance with the exercise program; (6) had visual and/or hearing impairments that could not be corrected with a lens and/or hearing aid; (7) were unable to communicate; or (8) were able to participate in other exercise programs.

### 2.2 Procedures

**Figure 1:**
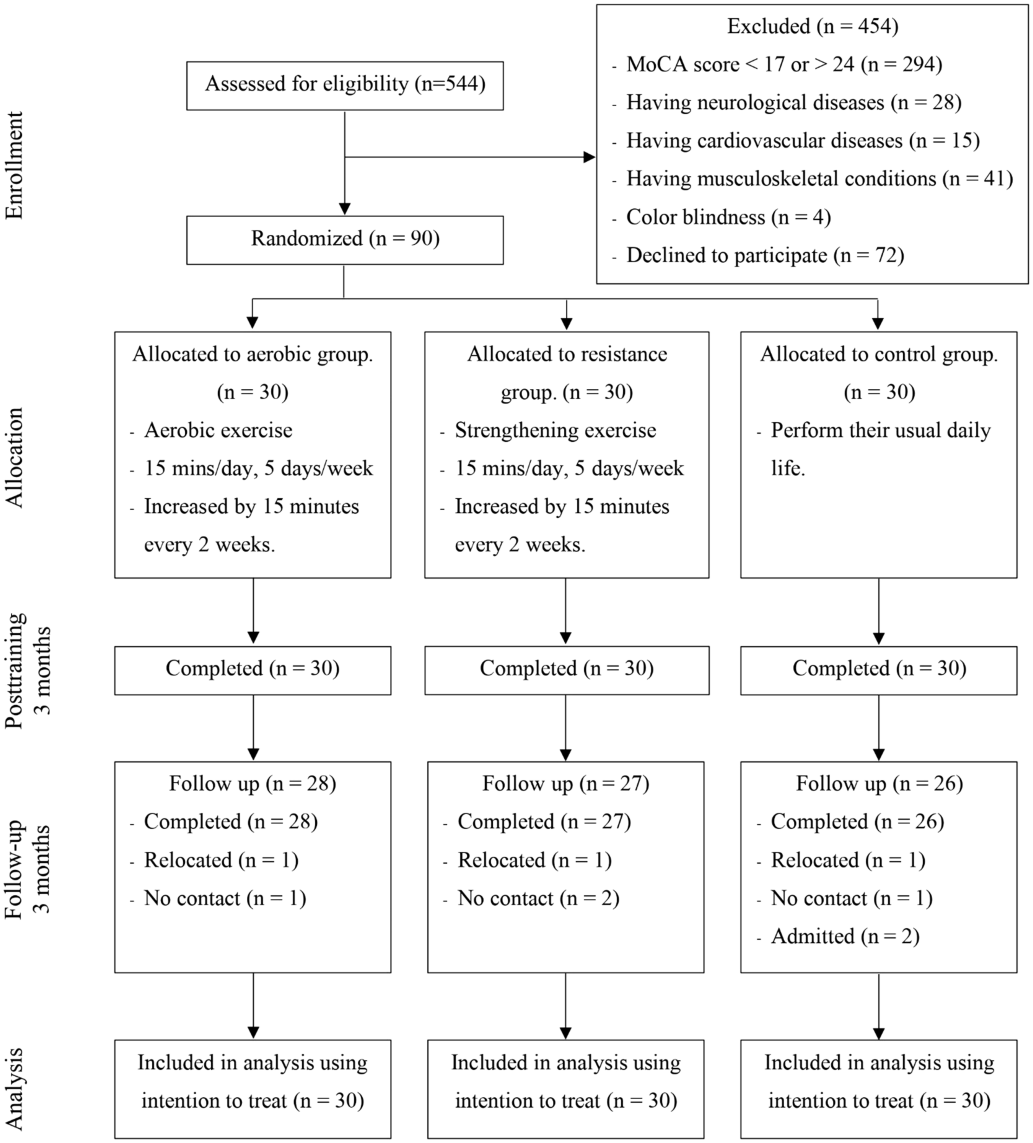
CONSORT flow diagram displaying the process of inclusion, allocation and attrition.

This research protocol was approved by the human ethics committee of the Physical Therapy Faculty, Srinakharinwirot University (PTPT2021-013) and was registered on the thaiclinicaltrials.org website (identification number: TCTR20231110003). The participants were randomly stratified by age and education level into three groups: (1) the aerobic exercise group, (2) the resistance exercise group, and (3) the control group (Figure 1). The allocation sequence was computer generated by an independent researcher who was not involved in instructing the exercise program and assessed the outcome measures and was sealed in an envelope before being given to the exercise program instructor. The low-intensity home-based exercises were designed to address safety concerns. These exercises were performed 5 days per week over a 3-month period, starting 15 min per day and increasing by 5 min every 2 weeks. Each exercise program consisted of a 10-min warm-up and 10-min cool-down.

### 2.3 Interventions

Participants in the aerobic group performed a home-based aerobic exercise program at low intensity with an exhaustion level of ≤13 points on the Borg scale (25), and they later progressed with increasing number of exercises, number of repetitions and complexity of the exercises (Table 1). The resistance group was subjected to a home-based resistance training program with an intensity appropriate to each participant’s fitness level, according to bodyweight, water bottles or items available at home, and subsequently progressed through increasing resistance, number of exercises, number of sets or repetitions and complexity of the exercises (Table 1). Before beginning their home-based exercises, participants in both groups received an explanation of the training program; this guaranteed that the exercises could be completed safely and correctly when performed alone at home, and each group received a different exercise handbook. The Borg Rating of Perceived Exertion scale was also explained so that participants could accurately rate themselves and as a recheck on their level of exercise intensity. This was to monitor and ensure that the exercise intensity remained low (with the Borg scale between 9 and 13), as required for safety in an elderly population with MCI. To record adherence to the exercise program, participants also received a logbook in which they could record their exercise. Additionally, the participants received weekly phone monitors to encourage exercise and inquire about any problems that might occur.

**Table 1:** Exercise program.

Weeks	Duration (mins/day)	Exercise groups	
		Aerobic exercise	Resistance exercise
1–2	15	Low impact exercise: indoor walk, march in place, step in difference directions Low intensity (≤13 point of Borg scale) *Example of Progression* 10 → 12 → 15 repetitions per exercise positions Add arm movement, squat while stepping	Shoulder flexion, abduction, elbow flexion, extension, hip extension, abduction, Knee extension, plantar flexion, wall push up, step ups Start 6–8 exercises 60 s rest between sets *Example of Progression* 2 × 10 → 2 × 12 → 2 × 15 repetitions per exercise positions Low loads → increase load when performance is good Add isometric contractions while exercising
3–4	20		
5–6	25		
7–8	30		
9–10	35		
11–12	40		

* Progression was adjusted every 2 weeks.

Participants in the control group were permitted to continue their usual daily life activities with the exception of engaging in any type of exercise or cognitive training until the end of the study. The participants received a logbook to record their daily routine, and they were checked by phone every week for any problems that might arise similar to those in the exercise groups.

### 2.4 Outcome measures

Outcome data were collected at pretraining, 3 months posttraining and 3 months after the end of the exercise program by assessors who were blinded to the group of participants and experienced in performing the measurements. The results of the neuropsychological assessments are listed in Table 2. All measurements were performed once for each test and in random order, with rest between tests.

**Table 2:** Clinical measurements and scoring in each category of outcome measure.

Category	Clinical measurement	Scoring
**Global cognitive function**	MoCA (24)	Score based on the number of the correct items and added 1 point for the participants who have less than 6 years of education
**Executive function**		
Mental flexibility	TMT-B (26)	Line alternating between numbers and Thai letters sequentially. Score based on the time of the completed task
Inhibitory control	SCWT (27)	(1) Read the word (name of the color), (2) name the color, and (3) name the color of words within 45 s/task. Score based on the number of the correct items and calculated the interference score using the formula; IG = CW - [(W x C)/(W + C)]
Working memory	DST-B (28)	Repeat the given set of numbers in the reverse order. Score based on the length of the set of numbers
**Attention**		
Processing speed	TMT-A (29)	Line connecting the numbers in order from 1 to 25. Score based on the time of the completed task
**Memory**		
Short-term memory	DST-F (28)	Repeat the given set of numbers in the same order. Score based on the length of the set of numbers
**Perceptual-motor function**		
Visuoconstructional reasoning	SDT (30)	Arrange the matches according to the example shown. Score based on the correctness of figures
**Physical mobility**	TUG-M (31)	After the command “go,” the participants stand up from sitting position, walk 3 meters, turn around, walk back to the chair, and sit down. Scores based on the time from “go” until the participant sat down

### 2.5 Statistical analysis

The data were analyzed using the statistical program IBM-SPSS version 22 for Windows. Descriptive statistics were used to describe the general characteristics of the participants. These analyses included all participants according to the intent-to-treat principle. Missing data at follow-up were replaced with posttraining data. The distribution of all the data calculated by the Kolmogorov–Smirnov test was not normally distributed, so the Kruskal–Wallis test was used for comparisons between the 3 groups at pretraining, posttraining and follow-up. The Wilcoxon signed rank test was used to compare the effects of exercise on cognitive and physical variables within groups, and the Mann–Whitney U test was used to compare the effects of exercise on cognitive and physical variables between groups. The statistical significance in this study was set at 0.05. Effect sizes were calculated to estimate the magnitude of differences in outcome variables between groups using the criteria 0.2, 0.5, and 0.8 as small, medium and large effects, respectively (32).

## 3 Results

The subject characteristics and pretraining assessments are presented in Table 3. A total of 90 subjects with an average age of 69.00 ± 5.03 years completed the 3-month exercise program. All 3 groups had similar characteristics, with the majority being female, having less than 6 years of education, doing housework, and having low physical activity levels (<600 MET minutes per week). The exception is for marital status, where most of the individuals in the resistance group were married but the majority of the individuals in the other groups were single. All the cognitive variables and physical variables were not different among the three groups at pretraining.

**Table 3:** Subject characteristics and pretraining neuropsychological assessments and physical performance.

	All participants (*N* = 90)		Aerobic group (*N* = 30)		Resistance group (*N* = 30)		Control group (*N* = 30)	
	*N*	%	*N*	%	*N*	%	*N*	%
Female	71	78.89	24	80.00	23	76.67	24	80.00
Marital status (marriage)	46	51.11	14	46.67	19	63.33	13	43.33
Education level (academic years ≤6)	48	53.33	16	53.33	16	53.33	16	53.33
**Employment status**								
Employment	16	17.78	6	20.00	5	16.67	5	16.67
Unemployment	15	16.67	5	16.67	3	10.00	7	23.33
Housework	59	65.56	19	63.33	22	73.33	18	60.00
**Level of physical activity (MET minutes per week)**								
Low (<600)	51	56.67	18	60.00	17	56.67	16	53.33
Moderate (600–1,500)	29	32.22	7	23.33	9	30.00	13	43.33
High (≥1,500)	10	11.11	5	16.67	4	13.33	1	3.33
	**Mean (SD)**		**Mean (SD)**		**Mean (SD)**		**Mean (SD)**	
Age (years)	69.00 (5.03)		68.60 (4.86)		68.70 (4.72)		69.70 (5.55)	
MoCA	20.06 (1.94)		20.17 (2.09)		19.60 (1.83)		20.40 (1.87)	
TMT-A (s)	71.32 (38.37)		60.01 (17.41)		77.50 (40.33)		76.45 (48.88)	
TMT-B (s)	199.88 (161.00)		174.95 (174.26)		185.64 (157.66)		239.04 (147.96)	
SCWT	−13.62 (6.98)		−13.66 (6.43)		−15.61 (6.10)		−11.59 (7.92)	
DST-F	6.23 (1.26)		6.27 (1.34)		5.93 (1.11)		6.50 (1.31)	
DST-B	2.86 (0.53)		2.90 (0.61)		2.77 (0.50)		2.90 (0.48)	
SDT	9.26 (1.50)		9.27 (1.53)		9.30 (1.47)		9.20 (1.56)	
TUG-M (s)	15.28 (3.88)		14.56 (3.02)		16.21 (4.32)		15.08 (4.13)	

### 3.1 Within-group effect

Participants in the aerobic and resistance groups showed high adherence to the exercise program (94.83 ± 3.48% and 96.67 ± 3.16%, respectively). Participants in the aerobic group reported an average exhaustion level of 11 points on the Borg scale, which was the same as that reported by those in the resistance group. The aerobic and resistance groups demonstrated significant improvements in global cognitive function, as measured by the MoCA, during posttraining, and these improvements were able to be sustained through follow-up (Table 4). With the exception of the stick design test (SDT), improvements in all specific cognitive functions were also evident in both the aerobic and resistance groups during posttraining and follow-up. In contrast, the control group did not show improvements in cognitive or physical performance at posttraining or follow-up.

**Table 4:** Comparison of changes in cognitive function domains and physical performance outcomes within groups at posttraining and follow-up.

Outcome measures	Aerobic group (*N* = 30)			Resistance group (*N* = 30)			Control group (*N* = 30)		
	Mean (SD)	% change	Effect size	Mean (SD)	% change	Effect size	Mean (SD)	% change	Effect size
**MoCA**									
Pretraining	20.17 (2.09)			19.60 (1.83)			20.40 (1.87)		
Posttraining	25.40 (2.54)	25.93	0.62^a^	24.53 (2.90)	25.15	0.61^a^	21.17 (2.77)	3.77	0.18
Follow-up	24.63 (2.67)	22.11	0.62^a^	23.47 (3.21)	19.74	0.59^a^	21.70 (3.56)	6.37	0.22
**TMT-A**									
Pretraining	60.01 (17.41)			77.50 (40.33)			76.45 (48.88)		
Posttraining	39.53 (10.70)	−34.13	−0.59^a^	45.83 (22.92)	−40.86	−0.62^a^	65.68 (36.37)	−14.09	−0.25
Follow-up	47.75 (16.42)	−20.43	−0.46^a^	50.91 (27.92)	−34.31	−0.54^a^	64.96 (31.78)	−15.03	−0.25
**TMT-B**									
Pretraining	174.95 (174.26)			185.64 (157.66)			239.04 (147.96)		
Posttraining	78.26 (34.08)	−55.27	−0.62^a^	88.26 (52.83)	−52.46	−0.62^a^	154.36 (92.64)	−35.43	−0.52^a^
Follow-up	88.55 (44.77)	−49.39	−0.62^a^	103.22 (68.97)	−44.40	−0.60^a^	160.48 (115.08)	−32.86	−0.60^a^
**SCWT**									
Pretraining	−13.66 (6.43)			−15.61 (6.10)			−11.59 (7.92)		
Posttraining	−0.38 (6.78)	97.22	0.62^a^	−4.64 (6.79)	70.28	0.62^a^	−12.25 (6.46)	−5.69	−0.06
Follow-up	−5.14 (8.85)	62.37	0.55^a^	−8.62 (7.75)	44.78	0.59^a^	−11.48 (6.13)	0.95	0.07
**DST-F**									
Pretraining	6.27 (1.34)			5.93 (1.11)			6.50 (1.31)		
Posttraining	7.90 (1.27)	25.99	0.61^a^	7.50 (1.20)	26.48	0.59^a^	6.53 (1.11)	0.46	0.05
Follow-up	7.07 (1.20)	12.76	0.48^a^	6.70 (1.32)	12.98	0.41^a^	6.60 (0.93)	1.54	0.06
**DST-B**									
Pretraining	2.90 (0.61)			2.77 (0.50)			2.90 (0.48)		
Posttraining	3.67 (1.09)	26.55	0.47^a^	3.40 (0.77)	22.74	0.46^a^	3.00 (0.59)	3.45	0.12
Follow-up	3.47 (1.14)	19.66	0.40^a^	3.17 (0.59)	14.44	0.38^a^	3.07 (0.52)	5.86	0.22
**SDT**									
Pretraining	9.27 (1.53)			9.30 (1.47)			9.20 (1.56)		
Posttraining	9.67 (1.49)	4.31	0.22	9.77 (1.63)	5.05	0.20	9.47 (1.41)	2.93	0.13
Follow-up	9.47 (1.17)	2.16	0.08	9.50 (1.31)	2.15	0.07	9.33 (1.40)	1.41	0.07
**TUG-M**									
Pretraining	14.56 (3.02)			16.21 (4.32)			15.08 (4.13)		
Posttraining	11.24 (1.79)	−22.80	−0.62^a^	11.94 (3.07)	−26.34	−0.62^a^	15.38 (5.55)	1.99	0.01
Follow-up	13.03 (3.73)	−10.51	−0.37^a^	13.66 (3.73)	−15.73	−0.44^a^	15.26 (5.79)	1.19	0.04

MoCA, Montreal Cognitive Assessment; SCWT, Stroop Color and Word Test; TMT, Trail Making Test; DST-F, Forward Digit Span Test; DST-B, Backward Digit Span Test; SDT, Stick Design Test; TUG-M, Timed Up and Go Test with manual task.

a Indicates statistical significance compared with the pretraining at *p* < 0.05, Negative values of TMT-A, TMT-B and TUG-M mean improvement.

### 3.2 Between-group effect

**Figure 2:**
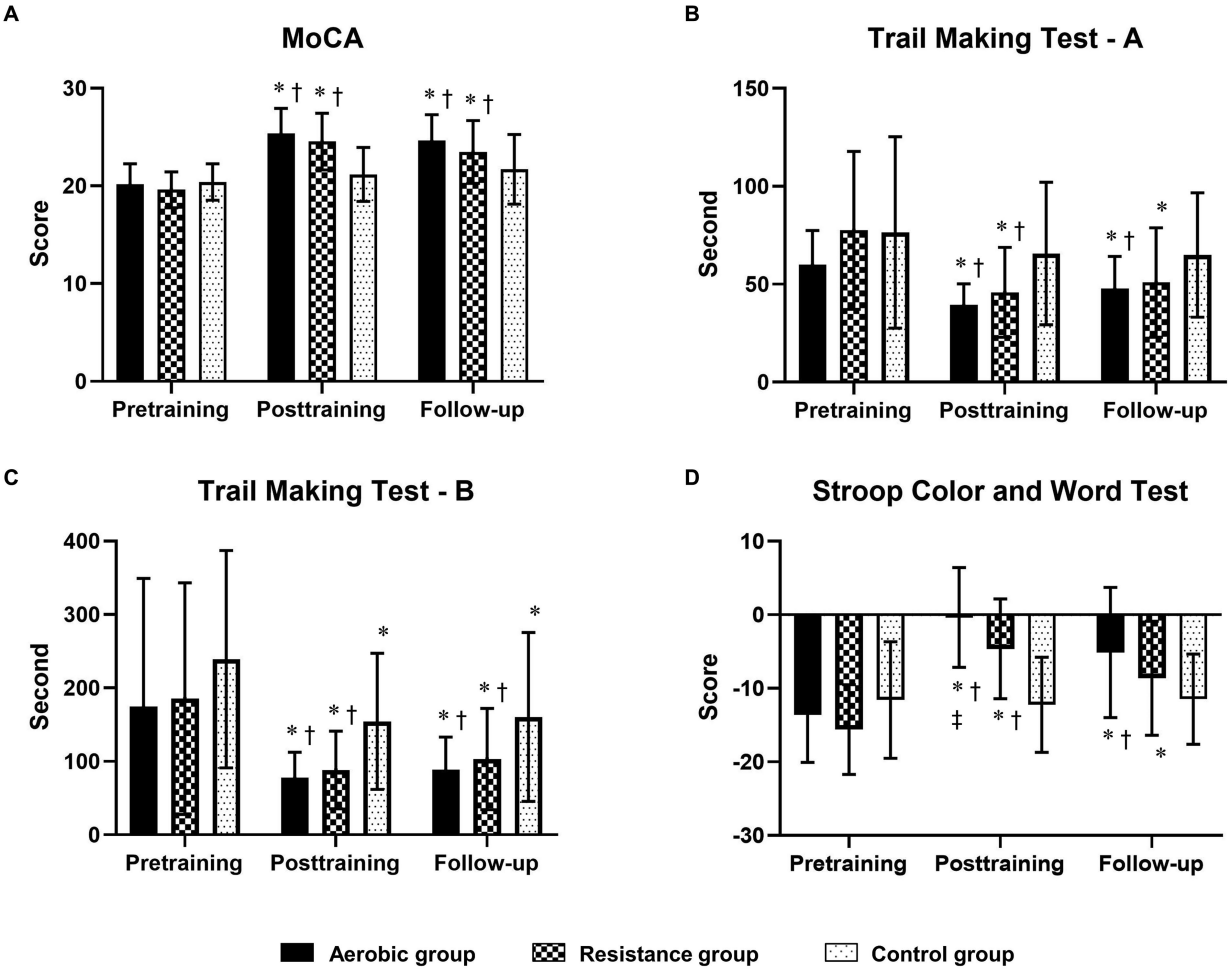
Comparison of changes in cognitive function domains [global cognitive function, processing speed, mental flexibility and inhibitory control measured by the MoCA **(A)**, the Trail Making Test-A **(B)**, the Trail Making Test-B **(C)** and the Stroop Color and Word Test **(D)**] between the 3 groups at posttraining and follow-up. The trail-making test **(A,B)** results are displayed in seconds, with shorter completion times indicating greater performance. * Indicates statistical significance compared within group at the pretraining at *p* < 0.05; † Indicates statistical significance compared with the control group at the same time at *p* < 0.05; ‡ Indicates statistical significance compared with the resistance group at the same time at *p* < 0.05.

**Figure 3:**
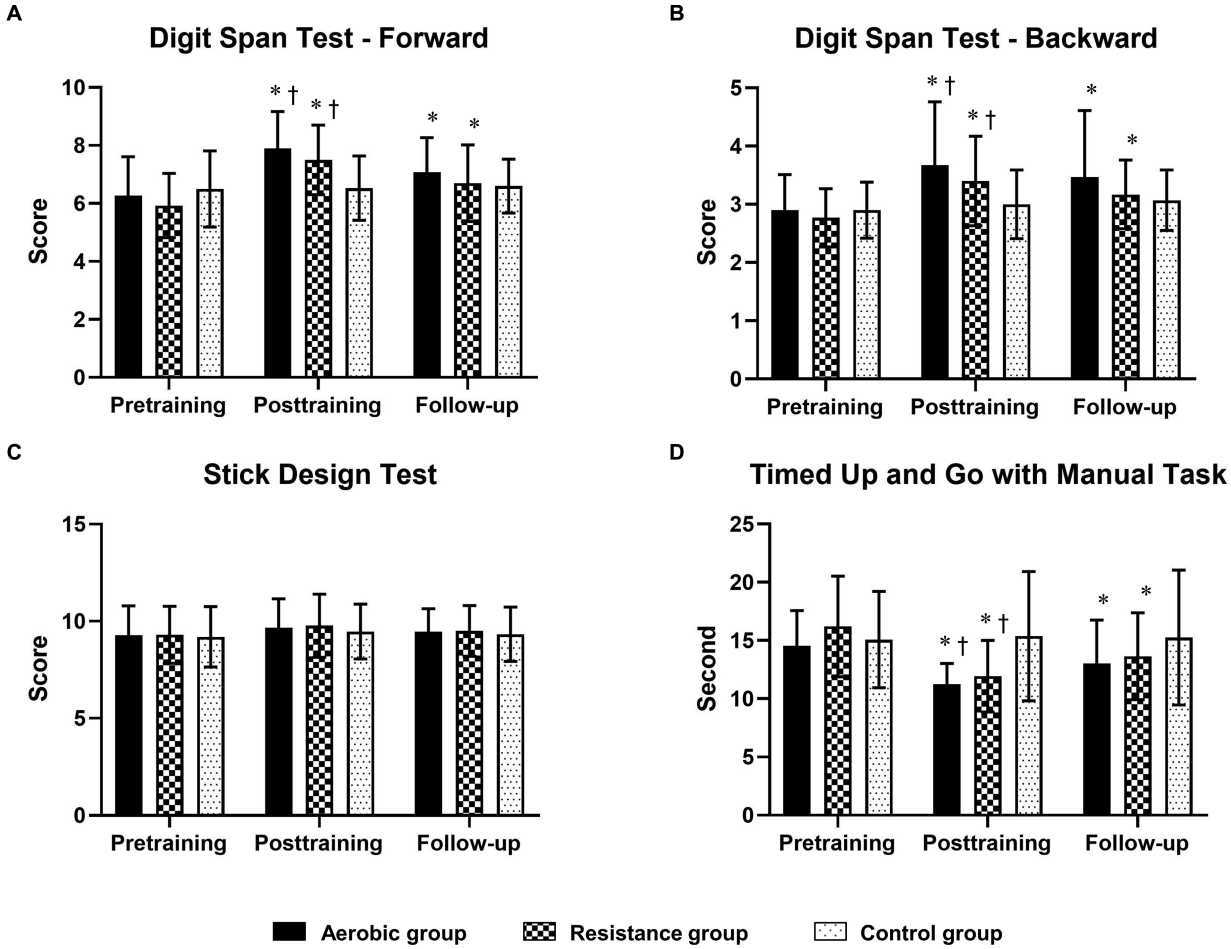
Comparison of changes in cognitive function domains [short-term memory, working memory and visuoconstructional reasoning measured by the digit span test—Forward **(A)**, digit span test—Backward **(B)** and stick design test **(C)**, respectively] and physical mobility [timed up and go test with manual task **(D)**] between the 3 groups at posttraining and follow-up. The timed up-and-go test with the manual task is displayed in seconds, with shorter completion times indicating greater performance. * Indicates statistical significance compared within group at the pretraining at *p* < 0.05; † Indicates statistical significance compared with the control group at the same time at *p* < 0.05.

Compared to those in the control group, the aerobic group differed significantly in terms of MoCA, TMT-A, TMT-B, and SCWT scores at posttraining and follow-up (Figure 2; Table 5). However, the DST-F, DST-B, and TUG-M scores of the aerobic group improved more than did those of the control group only after training (Figure 3). The resistance group had significantly better scores on the MoCA and TMT-B than did the control group at posttraining and follow-up, while the TMT-A, SCWT, DST-F, DST-B, and TUG-M differed from those of the control group only at posttraining. In contrast, there were no differences in cognitive or physical performance after training between the aerobic and resistance groups, except for inhibitory control, which was significantly better in the aerobic group than in the resistance group after training, as measured by the SCWT.

**Table 5:** Comparison of changes in cognitive function domains and physical performance outcomes between groups at posttraining and follow-up.

Outcome measures	Aerobic and control		Resistance and control		Aerobic and resistance		Effect size		95% CI	Effect size		95% CI	Effect size		95% CI
**MoCA**						
Posttraining	0.64*	[2.86, 5.61]	0.52*	[1.90, 4.83]	0.15	[−0.54, 2.28]
Follow-up	0.42*	[1.30, 4.56]	0.26*	[0.13, 3.52]	0.18	[−0.36, 2.70]
**TMT-A**						
Posttraining	−0.54*	[−40.22, −12.08]	−0.39*	[−35.62, −4.08]	−0.09	[−15.62, 3.03]
Follow-up	−0.30*	[−30.38, −4.05]	−0.30	[−29.52, 1.41]	−0.01	[−15.06, 8.74]
**TMT-B**						
Posttraining	−0.56*	[−112.62, −39.57]	−0.46*	[−105.29, −26.91]	−0.06	[−33.06, 13.06]
Follow-up	−0.35*	[−117.58, −26.27]	−0.27*	[−106.52, −7.99]	−0.08	[−44.83, 15.48]
**SCWT**						
Posttraining	0.68*	[8.45, 15.29]	0.51*	[4.19, 11.03]	0.29*	[0.76, 7.77]
Follow-up	0.41*	[2.39, 10.28]	0.24	[−0.75, 6.48]	0.21	[−0.83, 7.78]
**DST-F**						
Posttraining	0.50*	[0.75, 1.98]	0.38*	[0.37, 1.56]	0.17	[−0.24, 1.04]
Follow-up	0.22	[−0.09, 1.02]	0.04	[−0.49, 0.69]	0.14	[−0.29, 1.02]
**DST-B**						
Posttraining	0.38*	[0.21, 1.12]	0.29*	[0.05, 0.75]	0.10	[−0.22, 0.76]
Follow-up	0.19	[−0.06, 0.86]	0.11	[−0.19, 0.39]	0.09	[−0.17, 0.77]
**SDT**						
Posttraining	0.12	[−0.55, 0.95]	0.10	[−0.49, 1.09]	−0.03	[−0.91, 0.71]
Follow-up	0.03	[−0.53, 0.80]	0.05	[−0.53, 0.87]	−0.03	[−0.67, 0.61]
**TUG-M**						
Posttraining	−0.54*	[−6.30, −1.98]	−0.37*	[−5.77, −1.11]	−0.11	[−2.00, 0.61]
Follow-up	−0.23	[−4.75, 0.30]	−0.12	[−4.12, 0.93]	−0.12	[−2.56, 1.30]

DST, Digit Span Test; MoCA, Montreal Cognitive Assessment; SCWT, Stroop Color and Word Test; SDT, Stick Design Test; TMT, Trail Making Test; TUG-M, Timed Up and Go Test with manual task.

* Indicates statistical significance at *p* < 0.05.

## 4 Discussion

The uniqueness of this low-intensity home-based exercise study is that it enabled direct comparison of the effects of aerobic and resistance exercise programs on cognitive function in people with MCI. The results revealed that at 3 months posttraining, without between-group differences, both aerobic and resistance exercises improved physical performance as well as all cognitive domains, with the exception of the perceptual-motor function domain. This study also showed that such improvements could be maintained up to 3 months after training.

The exercise program in this study was classified as low intensity, as evidenced by the exhaustion level of 11 points on the Borg scale (33). The exercise programs in this study involved sufficient exercise volume adjusted to a high frequency and high total duration to induce changes in cognitive function. In our study, significantly large changes in SCWT, TMT-A, and TMT-B scores were observed in the aerobic and resistance exercise groups at 3 months posttraining. Using the minimal clinically important differences (MCIDs) of 9.3, 13.0, and 20.1 in the SCWT, TMT-A, and TMT-B, respectively (34), our changes in executive function (mental flexibility and inhibitory control) and attention (processing speed) reached clinical changes.

In comparison to those in the control group, the aerobic and resistance groups showed similar improvements in physical performance and all cognitive domains after training, except for visuoconstructional reasoning. The improvements in cognitive function from aerobic and resistance exercise were in line with previous findings. A previous study concluded that mild aerobic exercise intervention improved executive function, namely, inhibitory control, in older adults (35). Similarly, systematic reviews (8–10, 36) suggested that moderate-intensity aerobic exercise intervention increased global cognitive function, working memory and attention in older people with MCI. With regard to resistance exercise, the findings of this study were in accordance with those of several studies suggesting that resistance exercise has beneficial effects on global cognitive function (20), working memory (19) and processing speed (37) in older adults with MCI. Similarly, systematic reviews (16, 17) reported that resistance training improved global cognitive function, inhibitory control and short-term memory in adults.

The physiological mechanisms provide explanations for the impact of aerobic exercise on cognitive function. First, aerobic exercise leads to an increase in brain-derived neurotrophic factor (BDNF) (38, 39) resulting from the signaling of myokines, which are produced from muscles during exercise (40). Increasing BDNF levels resulting from aerobic exercise may result in improvements in cognitive and executive functions. Second, aerobic exercise reduces inflammatory cytokine levels (39), which are important predictors of MCI progression, and enhances physical fitness in older persons with MCI, which is linked to higher levels of BDNF and lower levels of inflammatory cytokines, leading to improved cognitive functions (41). Finally, aerobic exercise also promotes brain oxygenation during cortical activation of several regions, including the prefrontal cortex, which affects executive function (42).

Resistance exercise results in increased levels of serum insulin-like growth factor-1 (IGF-1), which is associated with cognitive function (43, 44). Low serum IGF-1 levels are correlated with impaired cognitive performance, especially a decrease in information processing speed in older adults (45). Therefore, improved cognitive function following resistance exercise may be explained by increased serum IGF-1 levels.

This study did not indicate improvements in visuoconstructional reasoning (as measured by the SDT) after aerobic or resistance exercise training. The lack of change in the SDT could be explained by the fact that this test specifically assessed fine motor function and eye-hand coordination. Both aerobic and resistance exercise programs involved large muscle groups and did not involve fine motor skills or eye-hand coordination. As a result, no distinct improvements in fine motor function or eye-hand coordination were observed in this study.

We performed a follow-up assessment at 3 months posttraining to assess whether the effect of training could be maintained. This study demonstrated that the aerobic group was able to maintain a positive impact on cognitive function in terms of global cognitive function, executive function (mental flexibility and inhibitory control) and attention (processing speed), whereas improvements in global cognitive function and mental flexibility were maintained in the resistance group. In addition, the aerobic group showed greater improvement in inhibitory control than the control group after training; this could be because aerobic exercise leads to an increase in circulating neurotrophin levels and gray matter volume in the prefrontal cortex as well as the preservation of neural connections between the prefrontal cortex and other regions of the brain (46, 47), which are responsible for inhibitory control (48). Moreover, resistance exercise increased gray matter density in the posterior cingulate cortex and increased functional connectivity among the posterior cingulate cortex, anterior cingulate cortex and hippocampus (49), which is implicated in decision-making based on action-outcome learning and related to memory (50).

To assess the prolonged effect of exercise training, Law et al. (10) reported that cognitive improvements in people with MCI were sustained for 12 weeks or more after the end of the intervention with ≥21 h of training. Similarly, our study revealed that these improvements were sustained after the exercise program for up to 3 months, with an average total training duration of 26 h. However, there are also differences in the intensity of exercise; for example, a previous study involved medium to high intensity exercise, while our study involved low intensity exercise.

### 4.1 Clinical implications and study limitations

The findings of this study highlight the importance of exercise in older adults, as recommended by the World Health Organization (WHO), the American College of Sports Medicine and the American Heart Association (51). Older adults should perform regular aerobic exercise and muscle strength training to maintain physical fitness and independence in daily life and reduce the risk of cognitive decline. According to the results of this study, both aerobic and resistance exercise had similar positive impacts on cognitive function. Therefore, older adults can choose either type of exercise to fit their own health conditions or exercise preference. For example, people at risk of cardiovascular disease may prefer aerobic exercise rather than resistance exercise to prevent them from holding their breath, which could increase the load on the cardiovascular system. Moreover, low-intensity exercise is considered an attractive exercise approach in terms of practicality and effectiveness. In addition, exercise encourages older people who are sedentary or have limitations and who are unable to exercise at a moderate intensity to continue receiving the benefit of exercise for improving cognitive function. Finally, home exercise provides an alternative for people with limited exercise space and who have travel difficulty, and this mode of exercise does not require specific exercise equipment.

There are several limitations in this study. The average age of the older adults who participated in this study was 69 years, so the results are limited and may not be generalizable outside of this age group. This study focuses solely on low-intensity exercise, a more direct comparison within the same study should be carried out to clarify the impact of different exercise intensities on cognitive function. Moreover, the results of this study were based only on cognitive changes; it is not known what the corresponding neurological changes or biological changes were. The underlying neurobiological mechanisms of low-intensity aerobic and resistance exercise should be investigated to better understand functional and/or structural changes in the brain.

## 5 Conclusion

Low-intensity aerobic and resistance exercise improved cognitive function in the areas of executive function, attention and memory domains in older persons with MCI, and these effects continued for another 3 months after training. The findings highlight the feasibility and accessibility of low-intensity exercise for older adults, which has an important impact on cognition.

## Data availability statement

The raw data supporting the conclusions of this article will be made available by the authors, without undue reservation.

## Ethics statement

The studies involving humans were approved by Ethical approval was received from the institutional review board of Srinakharinwirot University. The studies were conducted in accordance with the local legislation and institutional requirements. The participants provided their written informed consent to participate in this study.

## Author contributions

KK: Funding acquisition, Writing – review & editing, Writing – original draft, Methodology, Investigation, Formal analysis, Data curation, Conceptualization. NC: Writing – review & editing, Methodology, Formal analysis, Conceptualization. VS: Validation, Supervision, Writing – review & editing, Conceptualization. RB: Writing – original draft, Resources, Methodology, Investigation, Formal analysis, Data curation, Writing – review & editing, Validation, Supervision, Conceptualization.
